# Current Trends and Applications of PET/MRI Hybrid Imaging in Neurodegenerative Diseases and Normal Aging

**DOI:** 10.3390/diagnostics14060585

**Published:** 2024-03-10

**Authors:** Jonathan Lee, Jonathan Renslo, Kasen Wong, Thomas G. Clifford, Bryce D. Beutler, Paul E. Kim, Ali Gholamrezanezhad

**Affiliations:** 1 Department of Radiology, Keck School of Medicine, University of Southern California, Los Angeles, CA 90033, USA; 2 John A. Burns School of Medicine, University of Hawaii, Honolulu, HI 96813, USA

**Keywords:** PET/MRI, hybrid imaging, radiotracers, artificial intelligence, Alzheimer’s disease, dementia, aging

## Abstract

Dementia is a significant global health issue that is exacerbated by an aging population. Imaging plays an established role in the evaluation of patients with neurocognitive disorders such as dementia. In current clinical practice, magnetic resonance imaging (MRI) and positron emission tomography (PET) are primary imaging modalities used separately but in concert to help diagnose and classify dementia. The clinical applications of PET/MRI hybrid imaging in dementia are an active area of research, particularly given the continued emergence of functional MRI (fMRI) and amyloid PET tracers. This narrative review provides a comprehensive overview of the rationale and current evidence for PET/MRI hybrid dementia imaging from 2018 to 2023. Hybrid imaging offers advantages in the accuracy of characterizing neurodegenerative disorders, and future research will need to address the cost of integrated PET/MRI systems compared to stand-alone scanners, the development of new biomarkers, and image correction techniques.

## 1. Introduction

In recent years, the use of simultaneous positron emission tomography and magnetic resonance imaging (PET/MRI) hybrid imaging has transformed how we understand and image dementia and mild cognitive impairment (MCI). Its role is expected to expand further due to an anticipated increase in the population of elderly patients (greater than or equal to 65 years old). The prevalence of Alzheimer’s disease (AD) is approximately 4% in this population and increases dramatically with age [1]. This global health issue is also compounded by the underdiagnosis of dementia based on current clinical guidelines. Studies have shown a discrepancy between diagnosis rates and the projected increase in the population with dementia in developed and developing nations [2,3]. Ferri et al. summarized that age-specific dementia prevalence of AD should be expected to be similar worldwide, but differences exist due to difficulties establishing social impairment or lower survival [2]. Their team suggests that developing nations with a relatively low base number are expected to see rapid expansion. For example, they estimated that although in 2010 Latin American countries had approximately half as many people with dementia as North America, this number is expected to be in a similar range of 9 million by 2040.

Several bodies of work emphasize the importance of earlier intervention and prevention using common treatments such as acetylcholinesterase inhibitors or monoclonal antibodies against amyloid-beta (Aβ), which can be augmented by simultaneous PET/MRI imaging [4,5]. Amyloid deposition appears to plateau early in the presymptomatic or prodromal phases of AD, according to longitudinal data on individuals undergoing serial amyloid imaging and MR imaging examinations [6]. Individuals diagnosed with AD typically do not show a considerable increase in amyloid over time as determined by PET tracers, which highlights the role of hybrid imaging that can also detect subtle changes on MRI in the preclinical phases of dementia [7]. In addition, the recent introduction of novel anti-amyloid beta monoclonal antibodies represents a paradigm shift in the management of Alzheimer’s dementia, ushering in a new era of targeted therapies. Lecanemab, the first monoclonal antibody to be approved by the United States Food and Drug Administration, has been shown to significantly delay clinical decline and improve functional status among patients with Alzheimer’s dementia. However, the efficacy of lecanemab has only been established in the setting of early-stage disease. The detection of amyloid-beta deposition early in the course of illness is therefore of paramount importance. Advancements in PET/MR have the potential to identify patients with the earliest imaging signs of Alzheimer’s dementia who may benefit from lecanemab and similar anti-amyloid beta agents. Indeed, with the pressing demand to better understand and characterize dementia for this growing demographic, PET/MRI has a growing role in early diagnosis.

Previous review literature on hybrid PET/MR imaging in dementia was mostly limited to time periods before 2020 and has focused on various specifics such as changes in image acquisition settings, post-processing, regions of interest such as the hippocampus, and tracers [8,9,10]. As imaging continues to quickly improve, the purpose of this narrative review is to examine the latest articles published between 2018 and 2023 on the applications of PET/MRI in the characterization of various subtypes of dementia. This article will also summarize significant findings in key brain regions for its continued future use and the overall distribution of scanner field strengths, brands, and tracers in the latest set of studies.

## 2. Methods

A PubMed inquiry was performed with the MeSH terms (“PET-MR” OR “PET-MRI”) AND (“Dementia” OR “MCI” OR “Mild cognitive impairment” OR “Alzheimer” OR “AD”) on 20 January 2024 with dates restricted to between 2018 and 2023. Common abbreviations were employed to capture papers of interest related to the application of hybrid imaging to subtypes of dementia or MCI. The inclusion criteria used to filter papers for initial eligibility included published in English, sample size of at least ten human subjects, related to MCI or dementia, and employed hybrid PET/MRI imaging. This is a critical distinction since several studies may have listed PET/MRI as a keyword but imaged patients with either modality separately at different time points. Abstracts were also excluded if the objective of this study was to compare the performance of different PET tracers or were from a conference proceeding. Two researchers were assigned to each paper to filter the abstracts and titles using these guidelines. The papers were further divided into categories such as clinical, technical, or review based on the article type displayed on PubMed. Further data extraction was performed on the clinical studies to describe current trends, and each article was read by at least two researchers on the team. Downstream analysis was performed with R (Version 4.2.2, The R Foundation for Statistical Computing, Vienna, Austria) and the following packages: tidyverse (Version 2.0.0) and scales (Version 1.2.1). Figure generation was performed on Adobe Illustrator (Version 26.2.1, Adobe Inc., San Jose, CA, USA).

## 3. Results

**Figure 1:**
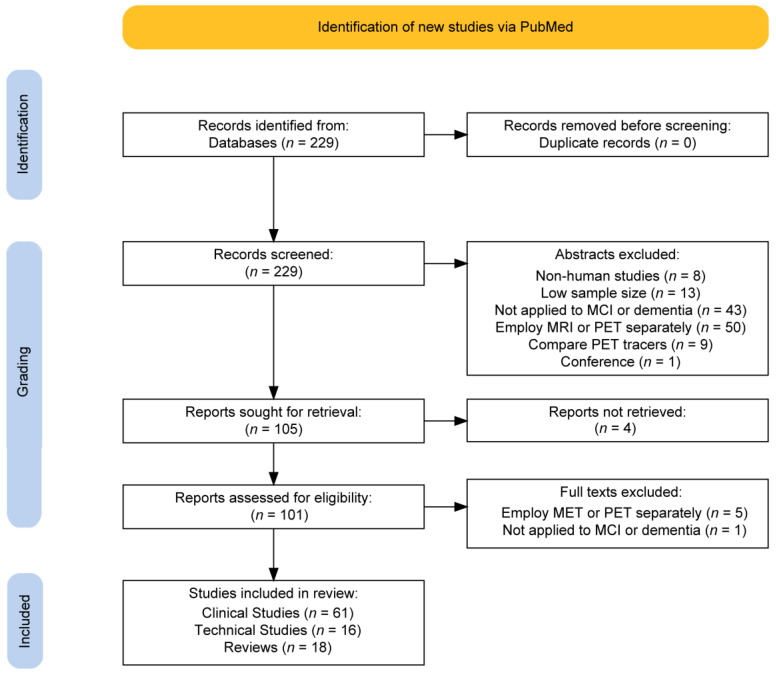
Flow chart summarizing the selection criteria of retrieved articles from a PubMed database search.

The literature search workflow is summarized in Figure 1. A total of 229 studies were initially identified using the PubMed search. After abstracts were excluded according to the inclusion criteria detailed in Section 2, the number of articles was narrowed to 105. The most common reasons for exclusion in the first screen were for employing MRI or PET separately (*n* = 50/229) and not being directly applied to the disease of interest (*n* = 43/229). The least common reasons were non-human studies (*n* = 8/229), low sample size (*n* = 13/229), comparing the performance of PET tracers (*n* = 9/229), and being a conference proceeding (*n* = 1/229). After attempting access with institutional subscriptions, 101 studies were obtained. After a full-text review, six more studies were removed due to not being applied to MCI or dementia directly or for employing MRI and PET separately. A total of 95 papers were included in this narrative review: 61 clinical, 16 technical, and 18 review manuscripts.

**Figure 2:**
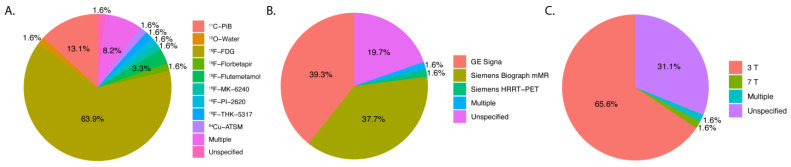
Pie charts demonstrating the distribution of key features of each clinical article that was filtered (*n* = 59). (**A**) PET tracers, (**B**) PET/MRI brand, and model (**C**) field strength. Note: Percentages may not sum to 100% due to label rounding.

**Figure 3:**
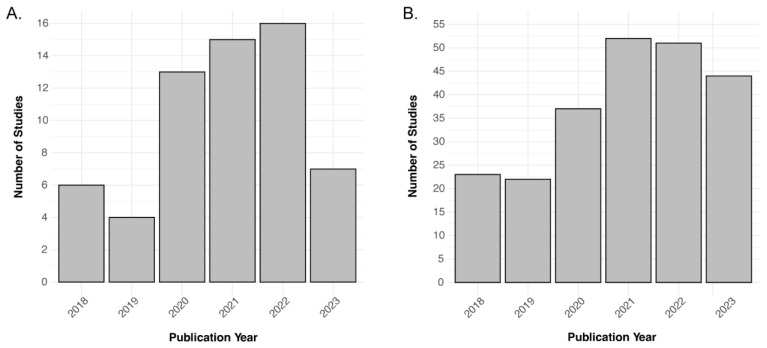
Bar charts showcasing the trend in the number of articles over the 2018–2023 data range. (**A**) *n* = 60 for the filtered articles and (**B**) *n* = 229 for the unfiltered database inquiry.

Figure 2 demonstrates the distribution of key characteristics in the trend of clinical studies (*n* = 60) in the past five years. The most commonly used PET tracer was ^18^F-FDG (63.9%; *n* = 39/61) with ^11^C-PiB (13.1%; *n* = 8/61) and ^18^F-Flutematamol (3.3%; *n* = 2/61) as runner-ups. ^15^O-Water, ^64^Cu-ATSM, ^18^F-THK-5317, ^18^F-PI-2620, and ^18^F-Florbetapir were each used in one study (1.6%; *n* = 1/61). A minority of studies had unspecified tracers (8.2%; *n* = 5/61). GE Signa was the most commonly used hybrid scanner (39.3%; *n* = 24/61), and the Siemens Biograph mMR was second (37.7%; *n* = 23/61). The most used field strength was 3 T (65.6%; *n* = 40/61), with only one at 7 T (1.6%; *n* = 1/61). A significant number of articles had unspecified field strengths (31.1%, *n* = 19/61). Figure 3 shows the trend in the number of articles over time related to the filtered and raw database query. The two years with the highest number of articles among the filtered articles were 2021 (24.6%; *n* = 15/61) and 2022 (26.2%; *n* = 16/61). Among the raw articles, 2021 (23.6%; *n* = 54/229) and 2022 (22.7%; *n* = 52/229) were also the years with the highest number of initially relevant articles. Both of these subplots in Figure 3 demonstrate an overall positive trend in the number of PET/MRI papers over time. Table 1 summarizes the main features of the eligible clinical studies.

## 4. Discussion

Overall, the number of reviews after filtering was 19.4% (18/93) of the eligible articles. Common themes across these review articles included the rise of artificial intelligence, technical improvements to PET/MRI imaging, and new and previous regions of interest for evaluating dementia [8,10,72,73,74,75,76,77]. The distribution of these article types remains consistent with this current review and is discussed further in the sections below along with additional insights from the clinical articles sourced in the past five years.

### 4.1. Artificial Intelligence

More in-depth discussions surrounding artificial intelligence (AI) and machine learning (ML) were present across all article types within this review [78,79,80]. Zhou et al. and Minoshima et al. summarized the common ML techniques used in radiology such as feed-forward neural networks (FFNN) and convolutional neural networks (CNN) [78,79]. This work showed that most AI studies in hybrid imaging employ a CNN or generative adversarial network (GAN) as a part of the classification model. CNNs are useful in automatically extracting spatial features from images and can learn relevant hierarchies, but are not effective in temporal information, which renders this modality ineffective for evaluating the progression of AD over time on volumetric data. GANs can generate synthetic data that can resemble real dementia samples; however, it can be challenging to train and can create unrealistic images. Logan et al. provide a similar explanation of different networks and explain the application of ensemble models which combine the output of different models for a final prediction [80]. Common themes in the review articles remain consistent with each other, such as brain regions of interest in dementia, but with the recent spike in interest in AI, there are more recent studies that discuss how ML improves PET/MRI acquisitions compared to previous periods. As it stands, CNNs typically have the best performance and potential when it comes to neuroimaging analysis and is the go-to model compared to other networks in this review. This model can automatically extract and learn image features and can efficiently analyze large datasets of PET/MRI images. Limitations of CNNs are not unique to this model and affect the field of AI as a whole such as sensitivity to imaging protocols, which can be mitigated by training on various scanners or field strengths, and overfitting if there is limited data. Within this discussion, the technical improvements and clinical classification of dementia subtypes aided by ML advancements will be further detailed.

### 4.2. Technical Improvements

#### 4.2.1. Attenuation Correction

Technical studies accounted for 19.4% (18/93) of the selected articles. A majority focused on accurate attenuation correction (AC) [39,81,82,83,84,85,86]. Traditionally, a density map for the correction had to be derived from MRI to properly measure the PET tracer activity but was challenging due to the lack of bone signal [87]. Several methods were studied to correct attenuation in a large cohort. Best methods, including RESOLUTE, a well-known segmentation-based AC method, were found to be sensitive to specified MRI sequences and thus could be significantly impacted by newer models or system updates [88]. Ladefegod et al. leveraged CNNs to convert non-attenuation corrected PET images to attenuation and scatter-corrected images, and due to the nature of neural networks, were shown to be more robust to different scanners [39]. Additional studies used deep learning to correlate a longitudinal relaxation rate, which is best evaluated by a T1-weighted study, with Hounsfield units in bone and soft tissues in the brain to perform AC [83]. Outside of ML, a study performed in 2020 by Sgard et al. used a zero echo-time (ZTE) MRI to generate the attenuation map and yielded lower bias and interindividual variability compared to a single-atlas AC method [81]. In addition, a quality assessment study by Øen et al. validated that MRI-based AC (MRAC) performed closely to CT-based AC (CTAC). Scores calculated for FDG uptake showed that the cerebellar region had the smallest mean absolute difference from this paper [82].

#### 4.2.2. Motion Correction

Various methods have been evaluated to correct motion. These range from mechanical methods, such as head restraints, to image-based methods, such as frame-based motion correction (FBMC), which registers the PET images to a reference position so that they are aligned [89]. Two technical articles in this review discussed and quantitatively measured the impact of motion correction on the imaging of dementia patients [90,91]. In a study performed by Tiss et al., using frame-by-frame motion detection reduced the standard deviation in the quantification of tau accumulation by 16% to 49% in areas such as the precuneus and amygdala [90]. Notably, in a study that included 30 subjects with dementia, Chen et al. found that motion correction had the greatest impact in regions with larger amplitude motion, such as the medial orbitofrontal cortex [91].

#### 4.2.3. Tracer and Imaging Techniques

A few articles within this review studied how to comprehensively evaluate the scores of tracer intensity on hybrid imaging [92,93]. One study by Coath et al. focused on using the Centiloid conversion to standardize measurements for amyloid-beta plaque accumulation measured by ^18^F-Florbetapir across different study cohorts, such as Insight 46 or the Alzheimer’s Disease Neuroimaging Initiative (ADNI) [92]. The standardized uptake value ratio (SUVR) is widely used for extracting data from PET images. This metric is dependent on many variables, including the tracer used, target and reference regions, and image acquisition parameters. The 0 Centiloids (CL) anchor point represents the average amyloid level in young healthy controls while 100 Centiloids correspond to the average level in a typical patient with AD with a generalized linear formula: Centiloids = a × (SUVR) + b. The coefficients are determined through a calibration process with samples on the anchor points. Another project by Ford et al. used a heuristic scoring system to count regions of the brain below a preset z-score threshold using statistical parametric mapping (SPM). This method standardizes and examines brain images on a voxel-by-voxel basis and employs statistical tests such as ANOVA or Student’s *t*-test to determine significant differences. They found that this scoring system had similar performance compared to the random forest method that was trained on all the z-scores to differentiate frontotemporal lobar degeneration (FTLD) from AD. Using this heuristic method is more accessible than a machine learning tool for labs that lack computational support.

Interestingly, there is an effort to generate zero-dose healthy baselines of FDG PET/MRI images and acquire hybrid imaging at ultra-low doses using GANs and CNNs to help the development of patient safety protocols [94,95,96,97]. Many clinicians interpret the FDG-PET component of hybrid images with healthy controls of a similar demographic. However, diminished tracer uptake is non-specific and may reflect intrinsic anatomical differences or represent sequela of prior neurologic insult, such as chronic microvascular ischemic change. Previous investigations have used variational autoencoders (VAEs), which are a type of neural network that learns to compress the input image data and then reconstruct it back into its original form. The variational component allows the encoder to distribute the input onto a latent space allowing for variability and more control over how the VAE generates a custom image. In one study by Hinge et al., these artificially generated images were compared with healthy controls and had invariance to mean relative percent difference and mean absolute value relative difference [94]. Another set of projects focused on preserving diagnostic quality with shorter scan times with a high-sensitivity time-of-flight (TOF) PET system. Behr et al. showed that the image quality on the TOF PET/MRI images was comparable to or better than PET/CT even with reduced scan times and established that the 4 min acquisition times received the highest scores. Although the brain and parotid glands did not show significant differences in SUV_max_, other normal tissues like the humerus, aorta, lung, spleen, and liver showed major differences. This suggests limitations in applying this technology beyond neuroimaging. In another study, ultra-low dose tau accumulation images were enhanced using a GAN [96]. The authors used 5% of the original full dose and found that the ultra-low-dose images had an underestimation in SUVRs in regions with classically higher uptake but that the bias was reduced in the enhanced images, especially in the amyloid-positive healthy population. This is especially useful in tracers that are specific for amyloid-beta plaques such as ^18^F-Florbetaben since they have a relatively short half-life, synergistically increasing the safety profile of these imaging runs [97]. These findings should be taken into consideration when designing future high-powered studies to reduce subject radiation dose.

Overall, ^18^F-FDG is the most prevalent tracer used in clinical practice and is the most represented among the articles extracted in this review. It is currently non-specific for AD pathology since hypometabolism can occur in various types of dementia and neurological conditions. However, the use of the tracer in both evaluation of AD and non-AD neurodegenerative conditions remains important since this tracer is widely used in clinical practice and has a wealth of literature for comparison. In addition, most of the tracers specific for amyloid and tau plaques such as ^18^F-Flutemetamol, ^18^F-MK-6240, and ^18^F-PI-2620 are primarily employed in research settings due to developing protocols. ^11^C-PiB has been well-validated and is highly regarded for its specificity for amyloid-beta plaques, but its short half-life of approximately 20 minutes requires an on-site cyclotron and limits its use in research institutions. On the other hand, ^18^F-Florbetapir is an agent that binds specifically to amyloid plaques and is approved for clinical use in many countries including the United States to estimate plaque density, making this tracer useful for AD evaluation. It is not approved, however, for confirming a diagnosis of AD. Our findings suggest that a combination of ^18^F-FDG and ^18^F-Florbetapir be employed in clinical practice for the characterization of AD, and a combination of ^18^F-FDG and ^11^C-PiB to be leveraged at research facilities, where available. For non-AD causes of dementia, ^18^F-FDG on its own could be appropriate as an initial imaging study in PET/MRI. Regarding variation across imaging techniques, it was shown in a meta-analysis that the accuracy of hybrid imaging markers is dependent on how the tracer is measured and the type of tracer itself [98]. Frisoni et al. stated that ^18^F-FDG had more variability compared to the amyloid biomarkers due to the lack of a strong consensus on imaging protocols and encouraged clinicians to analyze work using standard diagnostic criteria from the International Working Group and data analysis tools that are well validated such as FreeSurfer [98]. Consequently, PET/MRI performs best as long as there is a reference normative population such as healthy controls present for a baseline comparison within the same study.

### 4.3. Functional Connectivity

Functional connectivity studies, which revolve around associations between activities of different brain regions and often involve function MRI (fMRI), remain a critical area of focus in PET/MRI. Common networks studied in dementia include the default mode network (DMN), salience network (SN), central executive network (CEN), sensorimotor networks, as well as language and visual networks. The DMN is active when the brain is at wakeful rest, such as daydreaming and recalling memories, while the CEN is involved in high-level cognition, such as problem solving and decision making. The SN is critical for filtering relevant internal and external stimuli and coordinating responses to these stimuli. One team led by Zhang et al. showed that there was lower glucose metabolism observed in the DMN, CEN, and SN in patients with AD compared to the healthy controls [69]. This was further supported by studies that showed that the temporal properties of the DMN were altered in PET/MRI of patients with different stages of AD, meaning that affected patients spent a shorter amount of time in this network [53]. Specific disruptions in the DMN include a reduction in long-range functional connectivity between the medial prefrontal cortex (MPFC) and a decrease in the blood-oxygenation level-dependent (BOLD) functional connectivity within the precuneus of the DMN [32]. Within the SN, patients with posterior cortical atrophy and semantic dementia were shown to have decreased activity from the left superior frontal gyrus to the right anterior insula [21].

Another study by Carlson et al. found that white matter connectivity was impacted in the hippocampus by tau accumulation. This was measured with fractional anisotropy, finding that there were significant elevations in the entorhinal, perirhinal, and cornu ammonis (CA) 1 regions among patients with dementia or MCI. The limbic cortico-striato-thalmo-cortical (CTSC) circuit has been discussed in detail regarding the behavioral variant frontotemporal dementia in both the presymptomatic and symptomatic stages [42]. This analysis found that there was weakened frontostriatal connectivity but enhanced striatothalamic and thalamofrontal connectivity in asymptomatic carriers of a tau protein mutation compared to non-carriers. Common limitations within functional connectivity studies are that most are cross-sectional at a single time point (time of imaging), which does not show disease progression or the evolution of these network connectivity changes.

### 4.4. Brain Regions of Interest and Biomarkers

#### 4.4.1. Hippocampus

The hippocampus is of interest in hybrid imaging because this area is known to be important in memory and cognition, and volume losses have been associated with MCI and dementia. One study by James et al. found that patients with smaller hippocampal volume at age 70 had a faster search speed decline in the preceding 26 years, which was measured by the response time to identifying a target within a visual set-up [35,99]. For every 1 mL decrease in volume, there was an additional decline of approximately 1 point in search speed per year. Another body of work suggested that amyloid-beta patients with preclinical AD had a smaller presubiculum volume compared to the healthy controls [52]. However, not every study showed consistent results regarding the loss of whole-hippocampal volume. One group showed that although the whole-hippocampal SUV was not statistically different between AD/MCI and controls, there was a substantially lower SUV in the dentate gyrus (DG) in the test group compared to the controls [17]. Despite this variation, the hippocampus remains significant as an anatomical region of interest based on the papers included in this review, and we recommend that this area be evaluated in hybrid imaging studies for conditions along the spectrum of MCI and Alzheimer’s disease.

#### 4.4.2. Gray and White Matter

Studies examining gray and white matter features are critically important to the study of dementia in hybrid imaging since brain atrophy is a common feature in the disease group and the degree of volume differences can distinguish between subtypes of cognitive impairment. In one cross-sectional study, there were significant differences in white matter diffusion metrics such as fractional anisotropy (FA), radial diffusivity (RD), radial kurtosis (RK), and axonal water fraction (AWF) between the amyloid-beta positive- and -negative groups [25]. FA is a scalar value that quantifies the directionality of white matter and ranges from 0 to 1, where 0 represents equal diffusion in all directions while 1 means highly one-directional diffusion. Low values suggest that there is disruption to the fibers. RD is a measure of water diffusion perpendicular to the main axis of diffusion. Increased RD values suggest demyelination. Radial kurtosis characterizes the non-Gaussian behavior of water diffusion and an elevated value means that there is complex tissue microstructure such as axonal damage. Finally, AWF quantifies the fraction of water content associated with axonal space, and increases in AWF are typically associated with a loss of axons.

Outside of diffusion metrics, white matter hyperintensity volume (WMHV) may represent an important marker of dementia; one group found that greater volumes were associated with poorer tracing accuracy in a circle drawing task within patients that had a Familial Alzheimer’s Disease (FAD) mutation and sporadic Alzheimer’s [44]. Gray matter volume losses were also found to be weakly to strongly positively correlated with regional hypometabolism within the different lobes depending on the subtype of dementia. For example, there was moderately significant gray matter loss in the occipital lobes for dementia with Lewy bodies (DLB) and strongly significant gray matter loss in the superior parietal lobule in corticobasal degeneration (CBD) [27].

#### 4.4.3. Biomarker Evaluation

Iron, amyloid-beta, and tau protein accumulation assessments represent important neurodegenerative disease markers and have been used to track disease progression as well as the early detection of dementia. Amyloid and tau accumulation is the most common assessment performed in the evaluation of AD. A significant number of clinical studies within this review show an increased amyloid and tau protein burden in patients with different types of dementia [25,31,35,37,38,55,94]. These subtypes can be further differentiated depending on the local distribution of the amyloid. For example, in patients with early-onset familial AD, it has been shown that there is increased amyloid deposition in the striatum compared to late-onset sporadic AD [55]. Interestingly, amyloid-beta can be identified by tracers that directly bind to them such as Pittsburgh compound B (PiB). Iron plaques have also been shown to correlate to amyloid-beta plaques in specific regions such as the frontal and temporal cortex; however, iron levels alone were not directly linked to cognitive performance [62]. In addition, another study showed increased quantitative susceptibility mapping (QSM), a measure of iron accumulation, in the putamen in patients with AD compared with non-AD patients [61].

### 4.5. Associated Illnesses

There are numerous causes of cognitive impairment for which PET imaging may play an important role in diagnosis and management, including Parkinson’s disease (PD), normal pressure hydrocephalus (NPH), multiple sclerosis (MS), and Down’s syndrome. Within this review, one analysis of the characteristics of Parkinson’s disease with mild cognitive impairment (PD-MCI) showed that 8/25 patients (32%) had amyloid-beta positivity. The study identified significant deposition in regions like the right caudal and rostral middle frontal cortex, precuneus, and left pars triangularis in the amyloid-beta-positive patients [31]. A study on MS showed that lower myelin content as measured by decreased ^11^C-PiB uptake was associated with lower cognitive status [15]. For NPH, an investigation led by Mangalore et al. showed hypometabolism in the medial frontal and temporal lobes on PET and sulcal enlargement of the same areas on MRI, suggesting that the increased pressure on the brain alters perfusion and contributes to cognitive impairment [46]. Lastly, Down’s syndrome is unique in the fact that a large percentage of patients with this disease eventually develop cognitive impairment. One ML study used support vector machine (SVM) regression models to show that there was a significant reduction in white matter connectivity in PiB-positive patients with Down’s syndrome compared with PiB-negative patients [14]. Given the ongoing development in the literature for imaging protocols, there is no strong consensus for tracers in approaching other etiologies of dementia beyond AD. However, a similar approach to the one described in Section 4.2.3 can be potentially employed, in which ^18^F-FDG can be used for the initial study due to its prevalence and ^18^F-Florbetapir can be added on in clinical practice if there is suspicion of amyloid pathology among the dementia subtypes.

### 4.6. Scanners

It is critical for future recommendations to evaluate the most common field strengths, model brands, and tracers used in PET/MRI. The most commonly used tracer among the articles reviewed was FDG. 3 T was the most common field strength, which is consistent with the models currently available for commercial sale on the combined PET/MRI imaging systems market. GE and Siemens are the only two producers of integrated imaging systems, which is consistent with an almost even split between the majority of scanner brands extracted from this review.

Only one study within this review noted the advantages of using ultra-high field MRI in combination with PET on the hippocampus compared to the 1.5 T and 3 T strengths [22]. 7 T has been increasingly utilized to identify subtle findings that differentiate disease and physiologic brain states in the setting of Cushing’s disease, epilepsy, dementia, and similar clinical entities [100,101,102]. Choi et al. found that the 7 T integration provided a higher signal-to-noise ratio and stronger spatial resolution which enabled detailed segmentation of the hippocampal subregions that was not possible before. They found that there was significantly less metabolic activity in the middle CA1, posterior CA1, and CA2/3 regions of the left hippocampus and the middle and posterior CA2/3 regions of the right hippocampus in the AD group. This paper also described decreased activity between CA4 and the dentate gyrus (DG) within the hippocampus. Limitations include that 7 T has been mainly restricted to the research setting. However, 7 T MRI is expected to become widely available in the clinical setting in the near future and may allow for the detection of subtle hippocampal aberrations in pre-symptomatic stages of AD.

### 4.7. Comparison to MRI or PET

Advances in PET/MRI hybrid imaging including the development of new tracers and techniques have been fast-paced and increasingly and rigorously compared to PET or MRI alone. Historically, the first studies on PET/MRI were in the brain, combining PET detection of neurotransmitter activity with MRI detection of white matter damage [103,104,105]. A feasibility analysis performed by Schlemmer et al. in 2008 showed that image quality was comparable to the individual scanners, and the temporal and spatial registration allowed for accurate matching of tracer uptake and MR signal features [103]. Subsequent studies showed that leveraging features from both modalities led to stronger performance compared to each alone [36,106,107]. Dukart et al. in 2011 showed that a support vector machine (SVM) classification was able to discriminate between AD and frontotemporal lobar degeneration (FTLD) using gray-matter information on MRI and fluorodeoxyglucose (FDG) uptake on PET with an accuracy of 94%, which was higher than the model trained on MR information alone [106]. MRI on its own has been shown by prior studies to lack specificity in detecting AD, particularly in the early stages of the disease [108,109]. In a 2007 study by Matsunari et al., voxel-based morphometry (VBM) which is MRI-based had a specificity of 87% for early-onset AD compared to 96% in FDG-PET [108]. In addition, enthusiasm for PET/MRI grew with the increasing use of new tracers beyond FDG such as ^11^C-Pittsburgh compound-B (PiB). PiB was first introduced in 2004 and binds specifically to amyloid-beta plaques in the brain, allowing for direct quantification of plaque burden in the brain [110]. Certainly, these improvements may improve the specificity of multimodal image acquisition in dementia subtyping while enabling evaluation within a single session for many patients who may otherwise have difficulty tolerating multiple sessions.

PET/MRI has become more financially reasonable for the evaluation of cognitive impairment due to its increased efficacy [72,111]. Manuel et al. estimate that for every five-year delay in dementia onset, there are direct savings of approximately USD 8 billion per year or approximately USD 135,000 per person [112]. Prato et al. estimate savings of USD 115,000 per person assuming that PET/MRI costs approximately USD 2000 per study and approximately one in ten patients have evidence of cognitive impairment on hybrid imaging [72]. Currently, due to the slower-than-anticipated incorporation of hybrid systems, cycle times for newer generation of models may be greater than ten years. Due to this trend, more companies are aiming to build dedicated brain PET inserts that can augment stand-alone MRI systems, which is cheaper than a full hybrid system. Specifically, it costs approximately USD 6 million for a packaged hybrid system versus USD 1.3 million for a PET insert that can be added to a USD 3 million 3 T MRI system, making PET/MRI more attractive as a modality [72]. Increasing adoption of the technology will continue to motivate clinical studies and reviews to more strongly inform the direction of multimodal imaging.

### 4.8. Limitations

Limitations of this study include that PubMed indexing may require four to twelve weeks to complete for some journals. This may explain why the number of articles in 2023 from this literature search is lower compared to the previous two years. In addition, the narrative review focused on articles from PubMed-indexed articles, which reflects high-quality articles based on journal selection but does not include papers indexed in other databases such as Scopus and Web of Science. For describing macro-trends in the dataset and for an in-depth discussion of common themes, narrative reviews are typically sufficient in broad recommendations. However, potential areas of improvement are to expand the scope of the review systematically to encompass multiple databases and leverage a quality and bias assessment such as the Cochrane risk-of-bias tool [113].

### 4.9. Ongoing Challenges and Future Directions of Hybrid Imaging

Hybrid imaging will play a central role in the diagnosis of AD and other neurocognitive disorders. The early detection of amyloid-beta deposition is of profound importance in the emerging era of targeted monoclonal antibodies and other therapies. However, there remains a paucity of data about hybrid imaging for the diagnosis and management of FTD, LBD, and other major causes of cognitive decline. Indeed, in our comprehensive review, only eight studies included patients with a suspected diagnosis of FTD and only three included patients with clinical signs of LBD. The development of novel radiotracers and nuclear imaging techniques for FTD is an area of increasing interest; although relatively uncommon, FTD is often characterized by a young age of onset and rapid progression. Hybrid imaging may allow for earlier diagnosis and prompt initiation of neurobehavioral interventions for this rare but devastating condition. In addition, although I-123 ioflupane SPECT imaging and FDG PET/CT are frequently used to confirm a suspected diagnosis of LBD, sensitivity and specificity are somewhat limited. Hybrid imaging represents an opportunity to improve the evaluation of patients with clinical signs and symptoms of LBD. The diagnosis of other causes of neurocognitive impairment, including NPH and CBD, may also be improved with hybrid imaging, although further research is necessary to establish reliable imaging biomarkers for these conditions.

## 5. Conclusions

This review article summarizes PET/MRI studies from 2018 to 2023 and the advantages and disadvantages of simultaneous imaging within this period. This paper also describes recommendations for future use and the expected advances in hybrid imaging with the addition of new tracers and AI. Future directions of hybrid imaging include incorporating more AI techniques to improve the technical aspects of PET/MRI such as attenuation and motion correction, validating more multi-modal biomarkers to evaluate dementia, and elucidating more regions of interest and functional connectivity networks. Current challenges in the field include the increased cost of integrated PET/MRI systems compared to stand-alone scanners, the challenge of image registration and analysis of two layers of data, and the increased radiation exposure to patients. The existing data suggest that hybrid imaging will play a central role in the diagnosis, management, and monitoring of dementia. Additional research is necessary to establish hybrid imaging techniques for the evaluation of non-AD dementia and develop strategies to facilitate the timely initiation of pharmacologic and neurobehavioral interventions.

## Figures and Tables

**Table 1:** General characteristics of the clinical studies included in the review.

Title	Lead	Aims	Sample Size	Imaging	Journal	Year
Using simultaneous PET/MRI to compare the accuracy of diagnosing frontotemporal dementia by arterial spin labeling MRI and FDG-PET	Anazodo et al. [11]	Compare MRI-arterial spin labeling (MRI-ASL) with FDG-PET in diagnosing frontotemporal dementia (FTD)	10 FTD patients/10 HC	^18^F-FDG PET/MRI on 3 T Siemens Biograph	NeuroImage: Clinical	2018
The contribution of beta-amyloid to dementia in Lewy body diseases: a 1-year follow-up study	Biundo et al. [12]	Explore the contribution of Aß in Lewy body disease, especially in Parkinson’s disease patients with and without amyloid	40 PD patients (13 with dementia, 22 MCI, 5 normal) and 10 DLB patients	^18^F-Flutemetamol PET/MRI on 3 T Siemens Biograph mMR	Brain Communications	2021
Association between lower body temperature and increased tau pathology in cognitively normal older adults	Blessing et al. [13]	Cross-sectionally evaluate the association between body temperature (as a proxy for brain temperature) and tau pathology in cognitively normal older adults using plasma and CSF P-tau181 and PET-MR	21 patients	^18^F-MK-6240 PET/MRI	Neurobiology of Disease	2022
Support vector machine learning and diffusion-derived structural networks predict amyloid quantity and cognition in adults with Down’s syndrome	Brown et al. [14]	Assess the effectiveness of hybrid imaging to predict brain amyloid plaque burden, baseline cognition, and longitudinal cognitive change using support vector regression	95 patients with Down’s syndrome	^11^C-PiB PET/MRI on 3 T GE Signa	Neurobiology of Aging	2022
Myelin imaging measures as predictors of cognitive impairment in MS patients: A hybrid PET-MRI study	Campanholo et al. [15]	Highlight differences in myelin imaging modalities that can be used as predictors of cognitive dysfunction in patients with multiple sclerosis (MS)	51 MS patients/24 HC	^11^C-PiB PET/MRI on GE Signa	Multiple Sclerosis and Related Disorders	2022
Simultaneous FDG-PET/MRI detects hippocampal subfield metabolic differences in AD/MCI	Carlson et al. [16]	Study changes in FDG-PET and time-of-flight MRI (TOF-MRI) in Alzheimer’s disease, focusing on the hippocampus and its subregions	9 AD and 6 MCI patients/17 HC	^18^F-FDG PET/MRI on 3 T GE Signa	Scientific Reports	2020
Hippocampal subfield imaging and fractional anisotropy show parallel changes in Alzheimer’s disease tau progression using simultaneous tau-PET/MRI at 3 T	Carlson et al. [17]	Examine the development of tau pathology on PET and its relationship with hippocampal connectivity changes on diffusion tensor imaging (DTI)	5 AD and MCI patients/13 HC	^18^F-PI-2620 PET/MRI on 3 T GE Signa	Alzheimer’s & Dementia (Amsterdam)	2021
Direct prospective comparison of (18)F-FDG PET and arterial spin labeling MR using simultaneous PET/MR in patients referred for diagnosis of dementia	Ceccarini et al. [18]	Conduct a head-to-head comparison of FDG-PET and MRI-ASL in diagnosing cognitive impairment (Alzheimer’s disease [AD], Lewy Body Dementia [LBD], FTD) in patients with suspected cognitive impairment	27 suspected CI patients/30 HC	^18^F-FDG PET/MRI on GE Signa	European Journal of Nuclear Medicine and Molecular Imaging	2020
Evaluating the association between brain atrophy, hypometabolism, and cognitive decline in Alzheimer’s disease: a PET/MRI study	Chen et al. [19]	Evaluate changes in the hippocampus and default mode network as biomarkers for the diagnosis of AD	23 AD patients/24 HC	^18^F-FDG PET/MRI on 3 T GE Signa	Aging (Albany NY)	2021
Multiparametric hippocampal signatures for early diagnosis of Alzheimer’s disease using (18)F-FDG PET/MRI Radiomics	Chen et al. [20]	Explore the utility of hippocampal radiomic data to conduct early diagnosis of AD	51 AD patients and 55 aMCI patients/53 HC	^18^F-FDG PET/MRI on GE Signa	CNS Neuroscience & Therapeutics	2023
Characterizing Differences in Functional Connectivity Between Posterior Cortical Atrophy and Semantic Dementia by Seed-Based Approach	Chen et al. [21]	Demonstrate the existence of abnormal functional connectivity (FC) with an unaffected network in posterior cortical atrophy (PCA) and semantic dementia (SD) using neuropsychological assessments and PET/MRI scans	12 SD patients/11 HC	^18^F-FDG PET/MRI on 3 T GE Signa	Frontiers in Aging Neuroscience	2022
Glucose Hypometabolism in Hippocampal Subdivisions in Alzheimer’s Disease: A Pilot Study Using High-Resolution ^18^F-FDG PET and 7.0-T MRI	Choi et al. [22]	Compare the glucose metabolism of hippocampal divisions, a clinical diagnostic marker of AD, in mild-AD patients and healthy controls	9 eAD patients/10 HC	^18^F-FDG PET/MRI on 7 T Siemens HRRT-PET	Journal of Clinical Neurology	2018
Investigating the Roles of Anterior Cingulate in Behavioral Variant Frontotemporal Dementia: A PET/MRI Study	Chu et al. [23]	Explore the role that the anterior cingulate cortex plays in behavioral deficits and executive dysfunction using hybrid imaging	21 bvFTD patients/21 HC	^18^F-FDG PET/MRI on 3 T GE Signa	Journal of Alzheimer’s Disease	2021
Coupling relationship between glucose and oxygen metabolisms to differentiate preclinical Alzheimer’s disease and normal individuals	Ding et al. [24]	Explore the relationship between fMRI and FDG-PET signals in preclinical Alzheimer’s disease and Mild Cognitive Impairment (MCI), using biomarkers for early detection	15 CI and 20 preAD patients/27 HC	^18^F-FDG-PET/MRI and ^18^F-AV45-PET/MRI on 3 T GE Signa	Human Brain Mapping	2021
Diffusion MRI biomarkers of white matter microstructure vary nonmonotonically with increasing cerebral amyloid deposition	Dong et al. [25]	Cross-sectionally characterize the pathological white matter changes in AD and mild cognitive impairment (MCI) using integrated PET/MR imaging, kurtosis imaging (DKI), and axonal water fraction (AWF)	21 MCI or early AD patients/23 HC	^18^F-FDG PET/MRI on 3 T Siemens Biograph mMR	Neurobiology of Aging	2020
FDG PET/MRI for Visual Detection of Crossed Cerebellar Diaschisis in Patients With Dementia	Franceschi et al. [26]	To evaluate the presence of crossed cerebellar diaschisis in patients with suspected neurodegenerative disease	75 patients with neurodegenerative disease	^18^F-FDG PET/MRI on 3 T Siemens Biograph mMR	American Journal of Roentgenology	2021
Hybrid imaging in dementia: A semi-quantitative ((18)F)-fluorodeoxyglucose positron emission tomography/magnetic resonance imaging approach in clinical practice	Franceschi et al. [27]	Retrospectively assess the relationship between semi-quantitative changes in lobar-specific gray matter volumes and corresponding regions of brain fluorodeoxyglucose (FDG) hypometabolism in patients with dementia and neurodegenerative disease using FDG PET/MRI, NeuroQuant morphometric analysis, and z-scores	89 dementia patients	^18^F-FDG PET/MRI on 3 T Siemens Biograph mMR	World Journal of Nuclear Medicine	2020
((18)F)-Fluorodeoxyglucose positron emission tomography/magnetic resonance imaging assessment of hypometabolism patterns in clinical phenotypes of suspected corticobasal degeneration	Franceschi et al. [28]	Retrospectively evaluate the metabolic and volumetric abnormalities in patients with clinically suspected corticobasal degeneration (CBD) using FDG PET/MRI	75 suspected CBD patients	^18^F-FDG PET/MRI on 3 T Siemens Biograph mMR	World Journal of Nuclear Medicine	2020
Metabolic positron-emission tomography/magnetic resonance imaging in primary progressive aphasia and frontotemporal lobar degeneration subtypes: Reassessment of expected [(18)F]-fluorodeoxyglucose uptake patterns	Franceschi et al. [29]	Evaluate FDG uptake patterns in patients with frontotemporal lobar degeneration/primary progressive aphasia (FTLD/PPA) subtypes	51 FTLD/PPA patients	^18^F-FDG PET/MRI on 3 T Siemens Biograph mMR	World Journal of Nuclear Medicine	2021
Functional Abnormality Associated With Tau Deposition in Alzheimer’s Disease—A Hybrid Positron Emission Tomography/MRI Study	Fu et al. [30]	Investigate the characteristics of tau deposition and its impact on functional connectivity (FC) in AD using neuropsychological assessments and FDG PET/MRI scans	26 AD patients/19 HC	^18^F-THK5317 PET/MRI	Frontiers in Aging Neuroscience	2021
Quantification of Brain β-Amyloid Load in Parkinson’s Disease With Mild Cognitive Impairment: A PET/MRI Study	Garon et al. [31]	Study how Aβ affects clinical presentation and regional brain volumes in PD-MCI	25 PD-MCI patients	^18^F-FDG PET/MRI on 3 T	Frontiers in Neurology	2022
Reduced blood oxygenation level dependent connectivity is related to hypoperfusion in Alzheimer’s disease	Göttler et al. [32]	Investigate the relationship between functional connectivity in the posterior default mode network (pDMN) using resting-state fMRI blood oxygenation level-dependent signal fluctuations (BOLD-FC) and PET/MRI in patients with AD	42 AD patients/27 HC	^18^F-FDG PET/MRI on 3 T Siemens Biograph mMR	Journal of Cerebral Blood Flow and Metabolism	2019
The 100-plus Study of cognitively healthy centenarians: rationale, design and cohort description	Holstege et al. [33]	Cohort study identifying characteristics associated with escape/delay of cognitive decline in centenarians enrolled in the 100-plus Study using MMSE tests, PET/MRI or PET/CT, feces collection, and post-mortem brain donation	300 patients	Unspecified PET/MRI	European Journal of Epidemiology	2018
Neuroimaging, clinical and life course correlates of normal-appearing white matter integrity in 70-year-olds	James et al. [34]	Explore how changes in normal-appearing white matter relate to brain health and modifiable lifestyle factors, considering confounders such as sex, white matter hyperintensity volume, and ApoE4 status	502 patients	^18^F-Florbetapir PET/MRI on Siemens Biograph mMR	Brain Communications	2023
Adulthood cognitive trajectories over 26 years and brain health at 70 years of age: findings from the 1946 British Birth Cohort	James et al. [35]	Characterize individual changes in cognitive function through mid- and later life in relation to brain health measures using PET/MRI, amyloid PET, whole-brain volume, hippocampal volume, and white matter hyperintensity volume (WMHV)	468 patients	^18^F-FDG PET/MRI on 3 T Siemens Biograph mMR	Neurobiology of Aging	2023
Hybrid FDG PET/MRI vs. FDG PET and CT in patients with suspected dementia—A comparison of diagnostic yield and propagated influence on clinical diagnosis and patient management	Kaltoft et al. [36]	Retrospectively compare simultaneous FDG-PET/MRI with PET/CT in identifying cognitive impairment features, evaluating the clinical impact of adding MRI to CT	78 patients	^18^F-FDG-PET on 3 T Siemens Biograph mMR	PLoS One	2019
Prediction of Amyloid Positivity in Mild Cognitive Impairment Using Fully Automated Brain Segmentation Software	Kang et al. [37]	To score the predictive ability of regional volume information extracted from PET/MRI images using FreeSurfer for cerebral amyloid positivity in patients with MCI	130 aMCI patients	^11^C-PiB PET/MRI on 3 T Siemens Biograph mMR	Neuropsychiatric Disease and Treatment	2020
Assessment of Alzheimer’s Disease Imaging Biomarkers in World Trade Center Responders with Cognitive Impairment at Midlife	Kritikos et al. [38]	Analyze preliminary results of FDG-PET and amyloid PET scans in World Trade Center first responders, assessing the risk of dementia	6 CI and 6 MCI patients	^18^F-FBB-PET and ^18^F-FTP-PET on 3 T Siemens Biograph mMR	World Journal of Nuclear Medicine	2022
Estimation of brain amyloid accumulation using deep learning in clinical [(11)C]PiB PET imaging	Ladefoged et al. [39]	Develop a deep learning model for predicting SUVR and Amyloid Status in ^11^C PiB PET scans, using MRI in the test set	1309 CI patients	^11^C-PiB-PET/MRI	EJNMMI Physics	2023
Tau-PET imaging predicts cognitive decline and brain atrophy progression in early Alzheimer’s disease	Lagarde et al. [40]	Explore regional tau binding measured at baseline is associated with AD progression over 2 years using PET/MRI and CSF biomarkers	36 AD patients/15 HC	^18^F-FDG PET/MRI on 3 T	Journal of Neurology, Neurosurgery, and Psychiatry	2022
Reconfigured metabolism brain network in asymptomatic microtubule-associated protein tau mutation carriers: a graph theoretical analysis	Liu et al. [41]	Explore patterns of metabolism topology reconfiguration in microtubule-associated protein tau MAPT mutation carriers during presymptomatic stages of genetic frontotemporal dementia (FDT) using neuropsychological testing, genetic testing, and PET/MRI	32 bvFTD/33 HC	^18^F-FDG PET/MRI on 3 T GE Signa	Alzheimer’s Research & Therapy	2022
Altered metabolic connectivity within the limbic cortico-striato-thalamo-cortical circuit in presymptomatic and symptomatic behavioral variant frontotemporal dementia	Liu et al. [42]	Characterize the metabolic connectivity between areas of the limbic cortico–striatal–thalamic–cortical (CSTC) circuit in presymptomatic and symptomatic bvFTD patients	33 bvFTD patients/33 HC	^18^F-FDG PET/MRI on 3 T GE Signa	Alzheimer’s Research & Therapy	2023
Involvement of striatal motoric subregions in familial frontotemporal dementia with parkinsonism harboring the C9orf72 repeat expansions	Liu et al. [43]	Explore the roles of striatal motor subdivisions in the pathogenesis of parkinsonism resulting from C9ORF72 repeat expansions in patients with FTDP (frontotemporal dementia with parkinsonism) using PET/MRI and PET/CT	2 FTDP patients/17 HC	^18^F-FDG PET/MRI on 3 T GE Signa	NPJ Parkinson’s Disease	2022
Visuomotor integration deficits are common to familial and sporadic preclinical Alzheimer’s disease	Lu et al. [44]	Investigate the discernibility of subtle visuomotor deficits in familial and sporadic preclinical AD using β-amyloid-PET/MRI, whole-brain volume, and white matter hyperintensity volume in presymptomatic familial AD patients	31 FAD patients/390 HC	^18^F-FDG PET/MRI on 3 T Siemens Biograph mMR	Brain Communications	2021
Decoupling of regional neural activity and inter-regional functional connectivity in Alzheimer’s disease: a simultaneous PET/MR study	Maleki et al. [45]	Explore the effect of AD and MCI on the coupling between regional neural activity and inter-regional functional connectivity	33 AD patients/26 HC	^18^F-FDG PET/MRI on Siemens Biograph mMR	European Journal of Nuclear Medicine and Molecular Imaging	2022
Hydrocephalic Dementia: Revisited with Multimodality Imaging and toward a Unified Imaging Approach	Mangalore et al. [46]	Study the correlation between normal pressure hydrocephalus and dementia, analyzing imaging findings and clinical symptoms	13 NPH patients	^18^F-FDG PET/MRI	Journal of Neuroscience in Rural Practice	2021
Simultaneous resting-state FDG-PET/fMRI in Alzheimer Disease: Relationship between glucose metabolism and intrinsic activity	Marchitelli et al. [47]	Investigate how early AD-related local changes in resting-state glucose consumption relate to the changes in functional activity observed in stand-alone RS-fMRI studies	23 MCI or AD patients/23 HC	^18^F-FDG PET/MRI on 3 T Siemens Biograph mMR	NeuroImage	2018
Clinical utility of ^18^F-FDG-PET/MRI brain in dementia: Preliminary experience from a geriatric clinic in South India	Mukku et al. [48]	Further explore the use of hybrid imaging to subtype dementia in groups such as semantic-frontotemporal dementia and mixed AD	21 MCI patients	^18^F-FDG PET/MRI on Siemens Biograph mMR	Asian Journal of Psychiatry	2019
Multimodal analysis using [(11)C]PiB-PET/MRI for functional evaluation of patients with Alzheimer’s disease	Okazawa et al. [49]	Evaluate neurofunctional changes in dementia with multiple parameters (perfusion, SUV of ^11^C-PiB PET, neurofunctional connectivity using RS-fMRI) reflecting neurophysiologic activity	58 MCI patients/16 HC	^11^C-PiB PET/MRI on GE Signa	EJNMMI Research	2020
Cerebral Oxidative Stress in Early Alzheimer’s Disease Evaluated by (64)Cu-ATSM PET/MRI: A Preliminary Study	Okazawa et al. [50]	Evaluate the degree of oxidative stress and amyloid burden in the brains of AD patients	10 eAD patients/10 HC	^64^Cu-ATSM PET/MRI on 3 T GE Signa	Antioxidants (Basel)	2022
Noninvasive Measurement of [(11)C]PiB Distribution Volume Using Integrated PET/MRI	Okazawa et al. [51]	Explore the utility of a non-invasive method for determining the arterial concentration of radioactive tracer in PET image reconstruction	19 MCI patients/16 HC	^11^C-PiB PET/MRI on GE Signa	Diagnostics (Basel)	2020
Hippocampal subfield volumes and pre-clinical Alzheimer’s disease in 408 cognitively normal adults born in 1946	Parker et al. [52]	Cross-sectionally analyze the relationship between cerebral β-amyloid deposition and volume of individual hippocampal subfields in cognitively normal older adults using MMSE, PET/MRI	408 patients	^18^F-FDG PET/MRI on 3 T Siemens Biograph mMR	PLoS One	2019
Alterations in resting-state network dynamics along the Alzheimer’s disease continuum	Puttaert et al. [53]	Investigate the relationships between MEG and FDG PET/MRI signals, focusing on network connectivity and dynamics in Alzheimer’s disease and MCI	10 AD, 10 aMCI, and 10 SCD patients/10 HC	^18^F-FDG PET/MRI on 3 T GE Signa	Scientific Reports	2020
Decreased Alpha Peak Frequency Is Linked to Episodic Memory Impairment in Pathological Aging	Puttaert et al. [54]	Investigate the link between alpha brain activity and alterations in episodic memory as assessed by PET/MRI, FCSRT (Free and Cued Selective Reminding Test), and R-MEG (Resting-State Magnetoencephalography)	37 AD patients/19 HC	^18^F-FDG PET/MRI on 3 T GE Signa	Frontiers in Aging Neuroscience	2021
Prominent Striatum Amyloid Retention in Early-Onset Familial Alzheimer’s Disease With PSEN1 Mutations: A Pilot PET/MR Study	Qin et al. [55]	Investigate amyloid deposition in Early Onset Familial Alzheimer’s Disease (EOFAD) and its correlation with clinical symptoms	5 EOFAD and 15 AD patients/12 HC	^11^C-PiB-PET/MRI on Siemens Biograph mMR	Frontiers in Aging Neuroscience	2021
Alzheimer Disease and Mild Cognitive Impairment: Integrated Pulsed Arterial Spin-Labeling MRI and (18)F-FDG PET	Riederer et al. [56]	Compare PET/MR hypoperfusion and hypometabolism in patients with AD and MCI with healthy controls	45 AD patients and 20 MCI patients/11 HC	^18^F-FDG PET/MRI	Radiology	2018
Cerebral vasomotor reactivity across the continuum of subjective cognitive impairment, amnestic mild cognitive impairment and probable Alzheimer’s dementia: A transcranial Doppler and PET/MRI study	Saka et al. [57]	Study the relationship between cerebrovascular dysfunction and AD-associated neuronal degeneration	43 sMCI, aMCI, or probable AD patients	^18^F-FDG PET/MRI; ^18^F-Flutemetamol PET/MRI on 3 T GE Signa	Journal of Cerebral Blood Flow and Metabolism	2023
Effective connectivity in the default mode network is distinctively disrupted in Alzheimer’s disease-A simultaneous resting-state FDG-PET/fMRI study	Scherr et al. [58]	Investigate patterns of effective connectivity (EC) within the default mode network in patients with early AD using metabolic connectivity mapping (MCM) utilizing functional magnetic resonance imaging (fMRI) and FDG PET	35 eAD patients/18 HC	^18^F-FDG PET/MRI on 3 T Siemens Biograph mMR	Human Brain Mapping	2021
The impact of atlas-based MR attenuation correction on the diagnosis of FDG-PET/MR for Alzheimer’s diseases—A simulation study combining multi-center data and ADNI-data	Sekine et al. [59]	Assess the impact of vendor-provided atlases on FDG PET/MRI evaluation of AD	47 AD patients	^18^F-FDG PET/MRI on 3 T GE Signa	PLoS One	2020
Concordance of regional hypoperfusion by pCASL MRI and (15)O-water PET in frontotemporal dementia: Is pCASL an efficacious alternative?	Ssali et al. [60]	Help elucidate biomarkers on hybrid imaging that can differentiate frontotemporal dementia subtypes and psychiatric disorders	9 FTD patients/13 HC	^15^O-Water PET/MRI on Siemens Biograph mMR	NeuroImage: Clinical	2022
Quantitative susceptibility mapping in β-Amyloid PET-stratified patients with dementia and healthy controls—A hybrid PET/MRI study	Tiepolt et al. [61]	Study the amount of iron accumulation and Aβ in patients with AD using hybrid imaging	16 AD patients/10 non-AD patients/11 HC	^11^C-PiB PET/MRI on 3 T	European Journal of Radiology	2020
Simultaneous quantitative susceptibility mapping and Flutemetamol-PET suggests local correlation of iron and β-amyloid as an indicator of cognitive performance at high age	van et al. [62]	Investigate local correlations between iron and Aß plaque deposition and their impact on cognitive function	116 HC	^18^F-Flutemetamol PET/MRI on 3 T GE Signa	NeuroImage	2018
Spatial decrease of synaptic density in amnestic mild cognitive impairment follows the tau build-up pattern	Vanderlinden et al. [63]	Investigate tau distribution and its relation to synaptic density in amnestic mild cognitive impairment	12 aMCI patients/12 HC	^18^F-MK-6240 PET/MRI and ^11^C-UCB-J PET/MRI on 3 T GE Signa	Molecular Psychiatry	2022
In vivo synaptic density loss is related to tau deposition in amnestic mild cognitive impairment	Vanhaute et al. [64]	Study whether synaptic loss and neurofibrillary tangle load spatially overlap and correlate with the clinical presentation of patients with aMCI	10 aMCI patients/10 HC	^11^C-UCB-J PET/MRI and ^18^F-MK-6240 PET/MRI on 3 T GE Signa; ^11^C-PiB on Siemens Truepoint	Neurology	2020
A metabolism-functional connectome sparse coupling method to reveal imaging markers for Alzheimer’s disease based on simultaneous PET/MRI scans	Wang et al. [65]	Develop a metabolic connectome and functional connectome coupling method to explore how abnormal glucose metabolism and oxygen utilization relates to SCD and AD	54 SCD patients and 27 AD patients	^18^F-FDG PET/MRI on 3 T GE Signa	Human Brain Mapping	2023
Multiparametric imaging hippocampal neurodegeneration and functional connectivity with simultaneous PET/MRI in Alzheimer’s disease	Yan et al. [66]	To investigate the association between hippocampal neurodegeneration and MCI and AD	22 AD patients and 38 MCI patients/42 HC	^18^F-FDG PET/MRI on 3 T GE Signa	European Journal of Nuclear Medicine and Molecular Imaging	2020
Combining PET with MRI to improve predictions of progression from mild cognitive impairment to Alzheimer’s disease: an exploratory radiomic analysis study	Yang et al. [67]	Investigate if ^18^F-FDG PET/MRI could predict the progression of MCI into AD	377 MCI patients/94 HC	^18^F-FDG PET/MRI	Annals of Translational Medicine	2022
Brain Stem Glucose Hypermetabolism in Amyotrophic Lateral Sclerosis/Frontotemporal Dementia and Shortened Survival: An (18)F-FDG PET/MRI Study	Zanovello et al. [68]	Further investigate the presence of brain hypermetabolism in the brain and cervical spine cord of patients with amyotrophic lateral sclerosis/frontal temporal dementia continuum	28 ALS/FTD patients/13 HC	^18^F-FDG PET/MRI	Journal of Nuclear Medicine	2022
Disrupted coupling between salience network segregation and glucose metabolism is associated with cognitive decline in Alzheimer’s disease—A simultaneous resting-state FDG-PET/fMRI study	Zhang et al. [69]	Investigate the correlation between network segregation (functional connectivity) of core neurocognitive networks and glucose metabolism in cognitive decline	21 AD and 21 MCI patients/24 HC	^18^F-FDG PET/MRI on 3 T Siemens Biograph mMR	NeuroImage: Clinical	2022
Simultaneous PET/fMRI Detects Distinctive Alterations in Functional Connectivity and Glucose Metabolism of Precuneus Subregions in Alzheimer’s Disease	Zhang et al. [70]	Explore the involvement of the precuneus in Alzheimer’s disease, focusing on glucose metabolism and functional connectivity	20 AD and 23 MCI patients/27 HC	^18^F-FDG PET/MRI on Siemens Biograph mMR	Frontiers in Aging Neuroscience	2021
Changes of Metabolic Connectivity in Dementia with Lewy Bodies with Visual Hallucinations: A (18)F-Fluorodeoxyglucose Positron Emission Tomography/Magnetic Resonance Study	Zorzi et al. [71]	Examine the changes in neuronal activity between cortical regions in patients with visual hallucinations in dementia with Lewy bodies (DLB) using FDG PET/MRI scans and graph theory	26 DLB patients	^18^F-FDG PET/MRI on 3 T	Brain Connectivity	2021

Note: Healthy controls (HC) had normal cognition as measured by MMSE or alternate exam and were matched for factors such as age, gender, and education with the test cohorts.

## Data Availability

The data presented and analyzed in this study are available as Supplementary File S1.

## Supplementary Materials

The following supporting information can be downloaded at: https://www.mdpi.com/article/10.3390/diagnostics14060585/s1.

## Abbreviations

MRI	Magnetic Resonance Imaging
MRI-ASL	MRI-Arterial Spin Labeling
FDG-PET	Fluorodeoxyglucose Positron-Emission Tomography
FTD	Frontotemporal Dementia
HC	Healthy Control
Aß	Amyloid Beta
PD	Parkinson’s Disease
DLB	Dementia with Lewy Bodies
CSF	Cerebrospinal Fluid
P-tau181	Phosphorylated Tau at position 181
AD	Alzheimer’s Disease
MCI	Mild Cognitive Impairment
TOF-MRI	Time-of-Flight MRI
DTI	Diffusion Tensor Imaging
LBD	Lewy Body Dementia
CI	Cognitive Impairment
eAD	Early Alzheimer’s Disease
bvFTD	Behavioral variant Frontotemporal Dementia
preAD	Pre-Alzheimer’s Disease
DKI	Diffusional Kurtosis Imaging
AWF	Axonal Water Fraction
CBD	Corticobasal Degeneration
FTLD/PPA	Frontotemporal Lobar Degeneration/Primary Progressive Aphasia
FC	Functional Connectivity
PCA	Posterior Cortical Atrophy
SD	Semantic Dementia
aMCI	Amnestic Mild Cognitive Impairment
SCD	Subjective Cognitive Decline
BOLD-FC	Blood Oxygen Level-Dependent Functional Connectivity
MMSE	Mini-Mental State Examination
NPH	Normal Pressure Hydrocephalus
RS-fMRI	Resting-State Functional MRI
SUVR	Standardized Uptake Value Ratio
EOFAD	Early Onset Familial Alzheimer’s Disease
PD-MCI	Parkinson’s Disease with Mild Cognitive Impairment
FCSRT	Free and Cued Selective Reminding Test
R-MEG	Resting-State Magnetoencephalography
FAD	Familial Alzheimer’s Disease
WMHV	White Matter Hyperintensity Volume
ALS	Amyotrophic Lateral Sclerosis
